# Role of low-density lipoprotein electronegativity and sexual dimorphism in contributing early ventricular tachyarrhythmias following ST-elevation myocardial infarction

**DOI:** 10.3389/fcvm.2024.1285068

**Published:** 2024-03-04

**Authors:** Mei-Yao Wu, An-Sheng Lee, Yen-Nien Lin, Wei-Hsin Chung, Ke-Wei Chen, Chiung-Ray Lu, Yun-Fang Chen, Chia-Ming Chang, Wei-Chung Tsai, Yi-Tzone Shiao, Chu-Huang Chen, Kuan-Cheng Chang

**Affiliations:** ^1^ School of Post-Baccalaureate Chinese Medicine, China Medical University, Taichung, Taiwan; ^2^ Department of Chinese Medicine, China Medical University Hospital, Taichung, Taiwan; ^3^ Department of Medicine, Mackay Medical College, New Taipei City, Taiwan; ^4^ Division of Cardiovascular Medicine, Department of Medicine, China Medical University Hospital, Taichung, Taiwan; ^5^ School of Medicine, China Medical University, Taichung, Taiwan; ^6^ Graduate Institute of Biomedical Sciences, China Medical University, Taichung, Taiwan; ^7^ Division of Cardiology, Department of Internal Medicine, Kaohsiung Medical University Hospital, Kaohsiung, Taiwan; ^8^ Drug Development and Value Creation Research Center, Kaohsiung Medical University, Kaohsiung, Taiwan; ^9^ College of Medicine, Kaohsiung Medical University, Kaohsiung, Taiwan; ^10^ Center of Institutional Research and Development, Asia University, Taichung, Taiwan; ^11^ Vascular and Medicinal Research, Texas Heart Institute, Houston, TX, United States; ^12^ Institute for Biomedical Sciences, Shinshu University, Nagano, Japan

**Keywords:** low-density lipoprotein electronegativity, ST-elevation myocardial infarction, sudden cardiac death, ventricular fibrillation, ventricular tachycardia

## Abstract

**Background:**

Early ventricular tachycardia/fibrillation (VT/VF) in patients with ST-elevation myocardial infarction (STEMI) has higher morbidity and mortality. This study examines gender-differentiated risk factors and underlying mechanisms for early onset VT/VF in STEMI.

**Methods:**

We analyzed data from 2,964 consecutive STEMI patients between January 1, 2008 and December 31, 2021. Early VT/VF was defined as occurrence of spontaneous VT/VF of ≥30 s or requirement of immediate cardioversion/defibrillation within the first 48 h after symptoms. An ex vivo ischemic-reperfusion experiments were conducted in 8-week-old ApoE^−/−^ mice fed a high-fat diet to explore the underlying mechanisms of early VT/VF.

**Results:**

In 255 of out 2,964 STEMI patients who experienced early VT/VF, the age was younger (58.6 ± 13.8 vs. 61.0 ± 13.0 years old, *P* = 0.008) with a male predominance. The plasma levels of L5, the most electronegative subclass of low-density lipoprotein, was higher in early VT/VF patients compared to those without early VT/VF (*n* = 21, L5: 14.1 ± 22.6% vs. *n* = 46, L5: 4.3 ± 9.9%, *P* = 0.016). In the experimental setup, all male mice (*n* = 4) developed VT/VF post sham operation, whereas no such incidence was observed in the female mice (*n* = 3). Significantly, male mice exhibited considerably slower cardiac conduction velocity as compared to their female counterparts in whole heart preparations (25.01 ± 0.93 cm/s vs.42.32 ± 5.70 cm/s, *P* < 0.001), despite analogous action potential durations. Furthermore, isolated ventricular myocytes from male mice showed a distinctly lower sodium current density (−29.20 ± 3.04 pA/pF, *n* = 6) in comparison to female mice (−114.05 ± 6.41 pA/pF, *n* = 6, *P* < 0.001). This decreased sodium current density was paralleled by a reduced membrane expression of Nav1.5 protein (0.38 ± 0.06 vs. 0.89 ± 0.09 A.U., *P* < 0.001) and increased cytosolic Nav1.5 levels (0.59 ± 0.06 vs. 0.29 ± 0.04 A.U., *P* = 0.001) in male mice. Furthermore, it was observed that the overall expressions of sorting nexin 27 (SNX27) and vacuolar protein sorting 26 (VPS26) were significantly diminished in male mice as compared to female littermates (0.91 ± 0.15 vs. 1.70 ± 0.28, *P* = 0.02 and 0.74 ± 0.09 vs. 1.57 ± 0.13, *P* < 0.01, respectively).

**Conclusions:**

Our findings reveal that male STEMI patients with early VT/VF are associated with elevated L5 levels. The gender-based discrepancy in early VT/VF predisposition might be due to compromised sodium channel trafficking, possibly linked with increased LDL electronegativity.

## Introduction

Sudden Cardiac Death (SCD), primarily precipitated by sustained ventricular tachyarrhythmias, notably ventricular tachycardia/ventricular fibrillation (VT/VF), is a principal cause of mortality among patients diagnosed with ST-Elevation Myocardial Infarction (STEMI). Bearing the responsibility for a significant portion of premature deaths, surpassing any other individual cause, SCD continues to be a primary focus of public health initiatives. It has been approximated that early VT/VF, occurring within the initial 48 h post symptom onset, affects 4%–12% of STEMI patients, a proportion that significantly exceeds the incidence in NSTEMI patients (<1%) (1–3). Early VT/VF in STEMI can potentially lead to SCD, as well as augment the risk of in-hospital, short-term, or even long-term mortality (4–6).

In an effort to elucidate the incidence and risk factors for early VT/VF, a nationwide Danish study recognized several patient characteristics, significantly linked to elevated risks of VT/VF prior to primary percutaneous coronary intervention (PPCI). These risks remained significant even after adjusting for cardiovascular risk factors, infarct location, and thrombolysis in myocardial infarction flow. Independent risk factors comprised of an age less than 60 years, family history of sudden death, absence of preinfarction angina, usage of statins, history of atrial fibrillation (AF), and an alcohol intake exceeding 7 units weekly (2). Furthermore, a higher proportion of male patients was observed in the VF group as compared to the non-VF group during their first STEMI episode in this Danish demographic, a pattern consistent with the elevated incidence of SCD amongst men observed previously (7–9). Nonetheless, the pathophysiological mechanism accountable for these gender disparities in the incidence of early VT/VF in STEMI remains to be clarified.

Interestingly, the Danish investigation reported a positive correlation between statin therapy, indicative of elevated cholesterol levels, and VF according to the adjusted regression analysis. Similarly, Liu et al. established that dyslipidemia might heighten the risk of ventricular tachyarrhythmias during the acute phase (within 24 h) of STEMI (10). Conversely, a negative correlation between hypercholesterolemia and VF has been documented during the first incidence of STEMI (11). Unquestionably, additional comprehensive investigations are warranted to unravel the role of dyslipidemia or hypercholesterolemia in the predisposition to early VT/VF in STEMI, especially in the current scientific climate where we possess the capacity to analyze a variety of low-density lipoprotein (LDL) fractions, such as L5—the most electronegative component of LDL (12–14).

Given the enormous social and economic repercussions of VT/VF-induced SCD in younger male STEMI patients, it is of paramount importance to deepen our understanding of risk factors and mechanisms pertaining to gender-related susceptibility to early VT/VF SCD in these patients. Consequently, we aim to devise effective therapeutic strategies to counteract these fatal ventricular tachyarrhythmias. To this end, we embarked on a comprehensive analysis of the incidence and demographics of early VT/VF using a large case series comprising 2,964 STEMI patients admitted over a span of 14 years at a tertiary healthcare center. Additionally, we employed an ischemic-reperfusion animal study to investigate the underlying mechanisms.

## Methods

### Patient population

To evaluate the gender-specific link with early VT/VF in STEMI patients, we performed a retrospective examination of 2,964 STEMI patients from our Chest Pain Unit Database at China Medical University Hospital, spanning from January 1, 2008, to December 31, 2021. The STEMI diagnostic criteria were based on clinical manifestations, electrocardiography (ECG), or cardiac enzyme concentrations. ECG criteria for STEMI was defined as ST-segment elevation exceeding 1 mm in two contiguous limb leads or 2 mm in precordial leads, or by the emergence of new-onset left bundle branch block (15). We gathered clinical and laboratory data from the patients' electronic medical records in the database. Early VT/VF was characterized by a spontaneous onset of VT/VF lasting for 30 s or more, or requiring immediate cardioversion/defibrillation due to a hemodynamic compromise evidenced by consciousness disturbance or cardiogenic shock within the initial 48 h post symptom onset. Patients who developed VT/VF due to complications related to the PPCI procedure or secondary to profound shock or severe electrolyte or acid-base imbalance were excluded from the study.

### LDL isolation and quantification of L5

Plasma L5 levels were measured using a 30 ml peripheral blood sample obtained from an antecubital vein within 72 h in a subset of STEMI patients, both with and without early VT/VF, post-PPCI. Initially, LDL particles were isolated from patient samples through sequential potassium bromide density centrifugation, in order to eliminate other lipid components, including chylomicrons, very low-density lipoprotein, and intermediate-density lipoprotein fractions (16). Whole LDL was equilibrated via dialysis in a buffer A column, containing 20 mmol/L TrisHCl (pH 8.0), 0.5 mmol/L EDTA, and 0.01% NaN_3_. LDL samples were then separated via electrophoresis in 0.7% agarose. LDL subfractions, isolated based on electrical charge, were further separated and collected using fast protein liquid chromatography (GE Healthcare, Buckinghamshire, UK) with UnoQ12 anion-exchange columns (Bio-Rad Laboratories, Inc., Hercules, CA, US), as described previously (13).

### Animal preparations

Eight-week-old ApoE^−/−^ mice of both genders, residing in the Animal Resource Laboratory at China Medical University, were administered a high-fat diet (TestDiet 58Y1, TestDiet®, Richmond, IN, US) for eight weeks at the commencement of each experiment to stimulate atherogenesis. In the OVx group, female mice were ovariectomized as described previously (17). The mice were euthanized by 5% isoflurane (FORANE, ABBOTT Laboratories Ltd., UK) in a combination of oxygen and nitrous oxide mixed with air at a ratio of 50:50. Upon stabilization, the ovary were pulled out by grabbing the adipose tissue that surrounds the ovary in the abdominal cavity. The uterine horns were then ligated and the ovary were cut. Twenty-four hours after surgery, inject the animals subcutaneously with antibiotics and 5 mg/kg Rimadyl. For the E2 group, male mice were injected with 0.3 mg/kg/day 17β-estradiol during whole experimental period.

### Optical mapping

The experimental animals were administered with sodium pentobarbital (60 mg/kg) for anesthesia and heparin (200 U/kg) via intraperitoneal injection. Their hearts were promptly excised, cleaned with ice-cold phosphate-buffered saline, and cannulated through the aorta utilizing a 20-gauge blunt stainless steel needle. These hearts were subsequently perfused with Tyrode's solution containing, in mmol/L: 135 NaCl, 5.4 KCl, 1.8 CaCl_2_, 0.53 MgCl_2_, 0.33 NaH_2_PO_4_, 5.5 glucose, and 5 HEPES (pH 7.4) via a Langendorff setup, maintaining a constant flow. The perfusion pressure was continually tracked using PowerLab (ADInstruments), with flow rates adjusted as necessary to sustain perfusion pressures between 50 and 60 mmHg. The perfusion solution was aerated with a 95% O_2_/5% CO_2_ mixture and kept at 37 °C.

After a 10-min stabilization phase, hearts underwent acute regional ischemia in the apical and anterolateral left ventricle for 30 min. This was achieved by ligating the left anterior descending artery (LAD) immediately distal to its origin using a 6-0 silk suture with a loose double knot, forming a 2–3 mm diameter loop. A 2–3 mm long piece of PE-10 tubing was inserted into the loop parallel to the artery. The loop of the 6-0 silk ligature was gently tightened around the artery and tubing, and the ligature was secured in place with a slipknot. The cessation of blood flow through the LAD was confirmed by observing a color change in the anterior wall of the left ventricle. Subsequently, the slipknot in the 6-0 silk ligature was untied, and the PE-10 tubing was removed, allowing for a 2-h reperfusion of the hearts. The hearts were stained with 50 nmol/L Di-4-ANEPPS (Sigma-Aldrich, St. Louis, MO, US) for 7.5 min, then rinsed for 5 min. Mechanical motion was restrained via the administration of blebbistatin (5 μmol/L, Sigma-Aldrich). A pacing electrode was situated at the left ventricular apex for the initiation of ventricular tachyarrhythmias via programmed stimulation.

Bipolar pacing electrodes with an electronic stimulator (PowerLab, AD Instruments) were positioned in proximity to the central region of the anterior surface and the apex of the left ventricle. Burst ventricular pacing was initiated, starting with 8 bursts at a cycle length of 160 ms, followed by sequential bursts, each decreasing by a 10 ms decrement, until reaching a cycle length of 30 ms. Throughout these procedures, continuous monitoring for ventricular tachycardia (VT) was maintained, and any VT, whether induced or occurring spontaneously, was recorded during the 2-h reperfusion period. A run of VT was defined as 10 or more beats with a cycle length <100 ms. In the event of sustained VT or ventricular fibrillation (VF), the hearts underwent defibrillation.

The MATLAB Rhythm 2.0 software was utilized for the computation of conduction velocity (CV), action potential duration (APD), and APD dispersion. CV was determined from the isochronal maps employing the polynomial multi-vector method within a 5 × 5 pixel area (18). APD was calculated at 80% of repolarization (APD80). Activation maps were generated based on the activation time sequence, determined using the dV/dtmax definition. The average of all pixels across the entire heart was employed to calculate APD80, and the dispersion of APD80 was determined as the difference between maxAPD80 and minAPD80. Both CV and APD80 were measured at a pacing cycle length of 160 ms, with the analysis involving 5 beats per heart.

### Whole cell patch-clamp technique

Left ventricular cardiomyocytes, harvested from the experimental animals, were exposed to electrophysiological assessments. These tests were conducted utilizing the whole-cell patch-clamp technique with an Axon CNS 700B amplifier (Molecular Devices, CA, USA), Digidata 1550A data acquisition system, and pClamp software (Version 10, Molecular Devices) in the volatage clamp mode. Quiescent cells were placed in a chamber mounted on the stage of an inverted microscope (Eclipse Ti-U, Nikon Corporation, Japan) in bath solution containing (in mmol/L): 46 NaCl, 91 N-methyl-D-glucamine, 5.4 KCl, 1.8 CaCl_2_, 1.1 MgCl_2_, 6 HEPES, 22 glucose, and 0.33 NaH_2_PO_4_; pH was adjusted to 7.4 using NaOH. Cs^+^ (2 mM) and Co^2+^ (1 mM) were added to block potassium and calcium currents, respectively. Heat-polished glass electrodes (tip resistances about 2 MΩ when filled with pipette internal solution) were prepared from borosilicate glass capillaries (outer diameter 1.5 mm) by the Glass Microelectrode Puller (PC-10, Narishige International Inc., East Meadow, NY, USA). The internal solution contained (in mM) 130 CsCl, 30 TEA-Cl, 20 NaCl, 5 MgATP, 10 HEPES, and 0.2 Na_3_GTP; the pH was adjusted to 7.2 using CsOH at room temperature.

### Western blot

Heart tissues were homogenized using RIPA lysis buffer (Merck Millipore, Billerica, MA, USA), followed by determination of protein concentration using the Pierce BCA protein assay kit (Thermo Fisher Scientific, San Diego, CA, USA). Membrane and cytosolic protein fractions of cardiac tissue were isolated with a membrane extraction kit (Mem-PER™ Plus Membrane Protein Extraction Kit, Thermo Fisher Scientific) according to the manufacturer's recommendations. Protein concentrations were determined by the Pierce BCA protein assay (Thermo Fischer Scientific); absorbance was measured with the Infinite M1000 multifunctional monochromator-based microplate reader (TECAN, Männedorf, Switzerland).

Protein samples (50 μg each) were differentiated via SDS–polyacrylamide gel electrophoresis (SDS–PAGE; 10%) and subsequently transferred onto an immobilon®-P transfer membrane (Merck Millipore). The membranes were then subjected to blocking with 5% bovine serum albumin (BSA; Sigma-Aldrich) and subsequently probed with specific antibodies against targeted proteins and actin (SNX27, Nobus Biologicals, CO, USA, 1:1,000 dilution; VPS26, Thermo Fisher Scientific, 1:1,000 dilution; SCN5A, Thermo Fisher Scientific, 1:1,000 dilution; β-actin, GeneTex Inc., Irvine, USA, 1:2,000 dilution; Na pump, Thermo Fisher Scientific, 1:1,000 dilution; Connexin-43, Merck Millipore, 1:1,000 dilution). The detection of signals was accomplished using a chemiluminescence solution and subsequently captured with ChemiDoc-it 815 imaging system (UVP, Upland, CA, USA).

### Statistical analysis

Continuous variables adhering to a normal distribution are presented as mean ± standard deviation (SD). The Student's *t*-test was employed to assess differences between two groups. For the comparison of more than two groups, we utilized one-way analysis of variance (ANOVA) along with Tukey's *post hoc* analysis to account for multiple comparisons. Categorical data are indicated as *n* (%) and the Chi-square test was utilized to investigate differences therein. A two-sided *P*-value of less than 0.05 was considered to be statistically significant.

Multivariate logistic regression analysis was used to identify the independent risk factors for VT/VF in STEMI patients. Variables with a *P* < 0.05 on univariate analyses were considered to represent explanatory variables and were included in the multivariate analysis to identify the independent predictors of VT/VF. Sex, age, body mass index, current smoker, end-stage renal disease, chronic obstructive pulmonary diseases, estimated glomerular filtration rate, high-density lipoprotein, peak troponin I, and left ventricular ejection fraction were variables entered into a backward elimination logistic regression model. Backward elimination of variables was set to a significance level of 0.05 and based on the probability of the likelihood-ratio statistic and the maximum partial likelihood estimates. All statistical analyses were executed utilizing SPSS software version 12.0 (SPSS Inc., Chicago, IL, US). The present study obtained approval from our Institutional Review Board for the analysis conducted.

## Results

### Human subjects

To examine the gender-specific correlation of early VT/VF onset in STEMI patients, our study included 255 STEMI patients who developed early VT/VF and 2,709 STEMI patients who did not, all drawn from our Chest Pain Unit Database between January 1, 2008 and December 31, 2021. The average age of patients experiencing early VT/VF was found to be younger (58.6 ± 13.8 years vs. 61.0 ± 13.8 years, *P* = 0.008) and the male proportion was higher in this group (87.5% vs. 83.2%, *P* = 0.045) (Table 1). Baseline clinical characteristics and comorbidities such as diabetes mellitus, hypertension, hyperlipidemia, or previous stroke showed no significant differences between the two groups. However, higher percentages of patients with end-stage renal disease (5.4% vs. 2.6%, *P* = 0.015) and chronic obstructive pulmonary disease (4.9% vs. 2.1%, *P* = 0.037) were observed in the early VT/VF group. In patients who developed early VT/VF, a lower estimated glomerular filtration rate (60.3 ± 31.5 vs. 68.3 ± 29.1 ml/min per 1.73 m^2^, *P* = 0.023) and left ventricular ejection fraction (43.3 ± 13.3% vs. 50.8 ± 11.5%, *P* < 0.001) were recorded. Furthermore, this group displayed elevated peak troponin I levels (127.4 ± 174.1 vs. 68.0 ± 88.1 ng/ml, *P* < 0.001) and Killip class, compared to those without VT/VF. Consistently, there was a significant increase in the rate of in-hospital major adverse cardiac events (including non-fatal stroke, recurrent MI, targeted vessel revascularization, and death) in the early VT/VF group (30.6% vs. 6.7%, *P* < 0.001). The lipid profile metrics, encompassing total cholesterol, triglycerides, and low-density lipoprotein (LDL), showed no significant disparities between the two groups of patients. However, the high-density lipoprotein level was found to be lower in the VT/VF group (37.1 ± 12.6 vs. 39.5 ± 23.6 mg/dl, *P* = 0.021).

**Table 1:** Baseline characteristics of patients with VT/VF vs. no VT/VF.

Variables	VT/VF (*n* = 255)	No VT/VF (*n* = 2,709)	*P*-value
Age (year)	58.6 ± 13.8	61.0 ± 13.8	***0*** *.* ***008***
Men	223 (87.5)	2,254 (83.2)	***0***.***045***
Body mass index	25.4 ± 4.4	25.5 ± 13.8	0.896
Current smoker	119 (50.4)	1,192 (45.9)	0.103
Hypertension	112 (46.7)	1,356 (52.0)	0.064
Diabetes mellitus	76 (31.7)	776 (29.8)	0.659
Hyperlipidemia	21 (8.8)	328 (12.6)	0.202
Chronic kidney disease	13 (5.4)	127 (4.9)	0.672
End-stage renal disease	13 (5.4)	67 (2.6)	***0***.***015***
Chronic obstructive pulmonary diseases	7 (4.9)	100 (2.1)	***0***.***037***
Prevalent/incident atrial fibrillation	4 (1.7)	18 (0.7)	0.108
History of Cerebral vascular accident	6 (2.5)	105 (4.0)	0.158
ALT (U/L)	81.1 ± 104.2	63.1 ± 231.9	0.297
Creatinine (mg/dl)	1.8 ± 2.1	1.8 ± 6.7	0.716
eGFR (m/min per 1.73 m^2^)	60.3 ± 31.5	68.3 ± 29.1	***0***.***023***
hsCRP (mg/dl)	4.4 ± 7.1	3.4 ± 6.5	0.243
Na (mEq/L)	138.4 ± 4.1	136.7 ± 11.0	0.144
K (mEq/L)	3.8 ± 0.8	3.8 ± 0.6	0.685
Total cholesterol (mg/dl)	169.4 ± 56.7	173.7 ± 56.1	0.307
Triglyceride (mg/dl)	137.0 ± 164.7	148.7 ± 150.3	0.332
LDL (mg/dl)	112.1 ± 61.2	115.0 ± 41.0	0.501
HDL (mg/dl)	37.1 ± 12.6	39.5 ± 23.6	***0***.***021***
Peak troponin I (ng/ml)	127.4 ± 174.1	68.0 ± 88.1	***<0***.***001***
Culprit vessel			
Left anterior descending	206 (84.1)	2,219 (84.9)	0.799
Left circumflex	143 (58.6)	1,459 (55.9)	0.228
Right coronary artery	175 (71.7)	1,844 (70.6)	0.788
Killip class			***<0***.***001***
1	59 (26.0)	1,789 (71.8)	
2	19 (8.4)	282 (11.3)	
3	18 (7.9)	116 (4.6)	
4	131 (57.7)	302 (12.2)	
Left ventricular ejection fraction (%)	43.3 ± 13.3	50.8 ± 11.5	***<0***.***001***
In-hospital MACE	78 (30.6)	182 (6.7)	***<0***.***001***
Post-PCI TIMI flow			***0***.***030***
0	3 (1.6)	30 (1.4)	
1	2 (1.0)	6 (0.3)	
2	15 (8.1)	87 (4.1)	
3	165 (89.1)	1,985 (94.1)	

Data are expressed as the mean ± SD and *n* (%). ALT, Alanine Transaminase; eGFR, estimated glomerular filtration rate; HDL, high-density lipoprotein; hsCRP, high-sensitivity C-reactive protein; LDL, low-density lipoprotein; MACE, major adverse cardiac events; TIMI, thrombolysis In myocardial infarction; VT/VF, ventricular tachycardia/ventricular fibrillation.

The bold values indicates statistical significance.

Using multivariate logistic regression analysis, we identified that age (OR: 0.950, 95% CI: 0.926–0.975, *P* < 0.001), eGFR (m/min per 1.73 m^2^) (OR: 0.989, 95% CI: 0.978–0.999, *P* < 0.001), a past history of cardiovascular diseases (OR: 2.97, 95% CI: 1.73–5.11, *P* = 0.037), peak troponin I (ng/ml) (OR: 1.003, 95% CI: 1.001–1.005, *P* = 0.003), and left ventricular ejection fraction (OR: 0.970, 95% CI: 0.945–0.995, *P* = 0.019) were independent risk factors for STEMI (Table 2).

**Table 2:** Multivariate logistic regression: independent predictors for VT/VF in STEMI patients.

Variables	Multivariate analysis	
	OR (95% CI)	*P* value
Age	0.950 (0.926–0.975)	<0.001
eGFR (m/min per 1.73 m^2^)	0.989 (0.978–0.999)	0.037
Peak troponin I (ng/ml)	1.003 (1.001–1.005)	0.003
Left ventricular ejection fraction (%)	0.970 (0.945–0.995)	0.019

Variables entered on step 1: sex, age, body mass index, current smoker, end-stage renal disease, chronic obstructive pulmonary diseases, estimated glomerular filtration rate, high-density lipoprotein, peak troponin I, and left ventricular ejection fraction.

**Figure 1:**
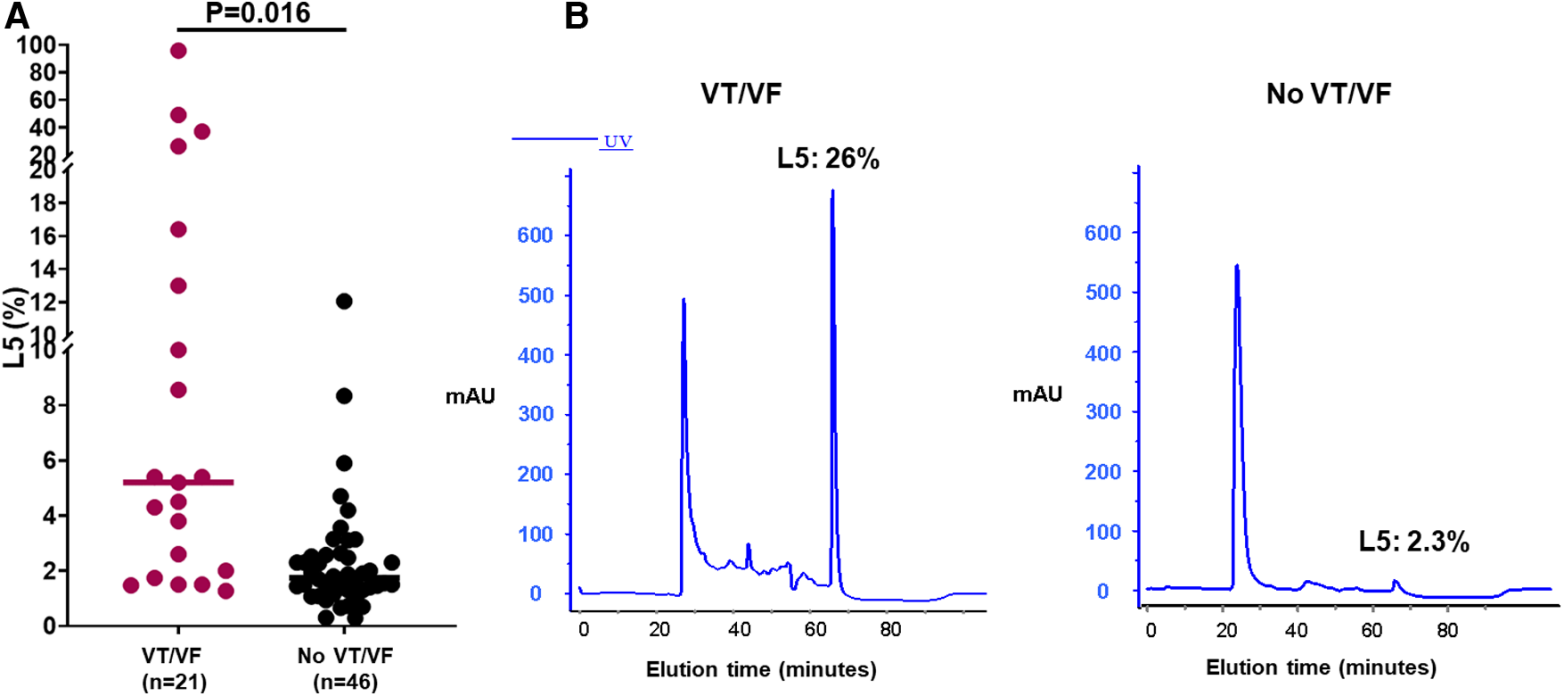
Assessment of L5 levels in a subset of patients with ST-elevation myocardial infarction (STEMI) with and without ventricular tachycardia/fibrillation (VT/VF). (**A**) The average plasma L5 concentration was notably elevated in STEMI patients experiencing early VT/VF (*n* = 21) compared to those devoid of early VT/VF (*n* = 46). The Student's *t*-test was employed to assess differences between two groups. (**B**) Illustrative instances of L5 levels in the groups with and without VT/VF. *X*-axis/minutes represents elution time.

In a subset of these STEMI patients, we conducted a comparative analysis of mean plasma L5 levels, collected within the initial three days post-presentation at the hospital (Table 3). No significant differences in age, gender, or comorbidities were observed between the groups with and without VT/VF. Patients experiencing early VT/VF exhibited elevated peak troponin I levels (88.02 ± 106.82 vs. 26.10 ± 23.12 ng/ml, *P* = 0.004) and lower left ventricular ejection fraction (LVEF) levels (41.97 ± 14.30% vs. 52.46 ± 14.84%, *P* = 0.044). It was observed that the average plasma L5 level was considerably elevated in STEMI patients who developed early VT/VF, as compared to those without early VT/VF (*n* = 21, L5: 14.1 ± 22.6% vs. *n* = 46, L5: 4.3 ± 9.9%, *P* = 0.016) (Figure 1).

**Table 3:** Baseline characteristics of patients with VT/VF compared to those without VT/VF in individuals undergoing L5 measurement.

Variables	VT/VF (*n* = 21)	No VT/VF (*n* = 46)	*P*-value
Age (year)	53.27 ± 7.38	54.78 ± 11.30	0.678
Men	21 (100)	43 (91.3)	0.301
Body mass index	31.05 ± 10.85	27.28 ± 6.92	0.163
Current smoker	13 (61.9)	29 (63.0)	1.000
Hypertension	9 (42.9)	27 (58.7)	0.294
Diabetes mellitus	6 (28.6)	16 (34.8)	0.781
Hyperlipidemia	2 (9.5)	5 (10.9)	1.000
Chronic kidney diseases	0 (0)	2 (4.3)	0.563
End-stage renal disease	0 (0)	1 (2.2)	1.000
Chronic obstructive pulmonary diseases	0 (0)	1 (2.2)	1.000
Prevalent/incident atrial fibrillation	0 (0)	0 (0)	1.000
History of Cerebral vascular accident	0 (0)	0 (0)	1.000
Total cholesterol (mg/dl)	162.7 ± 43.62	186.56 ± 43.90	0.131
Triglyceride (mg/dl)	154,09 ± 97.33	179.85 ± 170.11	0.635
LDL (mg/dl)	95.19 ± 43.71	114.95 ± 37.39	0.142
HDL (mg/dl)	45.52 ± 23.74	39.42 ± 12.51	0.258
Peak troponin I (ng/ml)	88.02 ± 106.82	26.10 ± 23.12	***0*** *.* ***004***
Culprit vessel			
Left anterior descending	18 (85.7)	33 (71.7)	0.240
Left circumflex	8 (38.1)	23 (50.0)	0.434
Right coronary artery	11 (52.4)	33 (71.7)	0.167
Killip class			**<0**.**001**
1	0 (0)	40 (87.0)	
2	2 (9.5)	1 (2.2)	
3	2 (9.5)	0 (0)	
4	17 (81.0)	5 (10.8)	
Left ventricular ejection fraction (%)	41.97 ± 14.30	52.46 ± 14.84	***0***.***044***
In-hospital MACE	0 (0)	0 (0)	1.000
Post-PCI TIMI flow			0.622
0	0 (0)	1 (2.2)	
1	2 (9.5)	1 (2.2)	
2	1 (4.8)	1 (2.2)	
3	18 (85.7)	43 (93.4)	

Data are expressed as the mean ± SD and *n* (%). ALT, Alanine Transaminase; eGFR, estimated glomerular filtration rate; HDL, high-density lipoprotein; hsCRP, high-sensitivity C-reactive protein; LDL, low-density lipoprotein; MACE, major adverse cardiac events; TIMI, thrombolysis In myocardial infarction; VT/VF, ventricular tachycardia/ventricular fibrillation.

The bold values indicates statistical significance.

### Animal model and laboratory experiments

**Figure 2:**
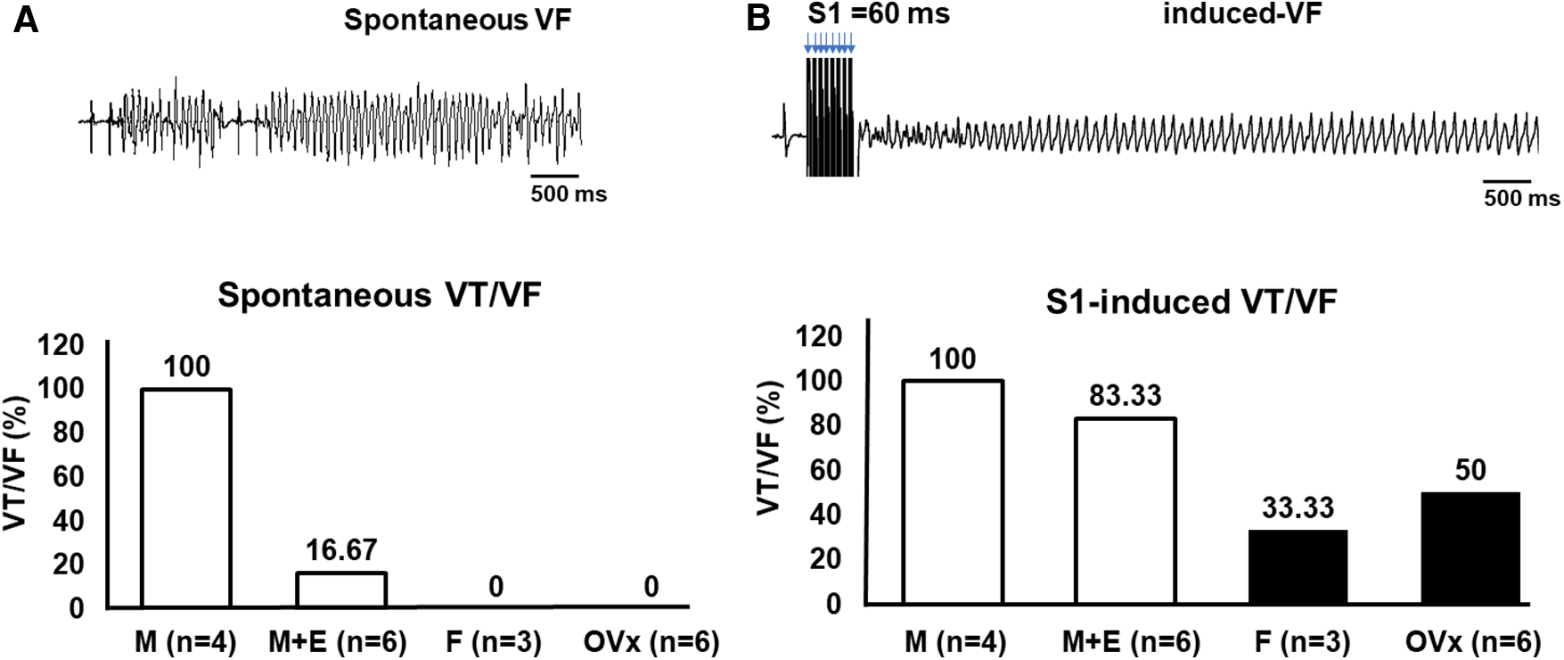
Manifestations of spontaneous or provoked VT/VF in ex vivo ischemia-reperfusion mouse model. (**A**) The incidence rate of spontaneous VT/VF was highest in sham-operated male mice (*n* = 4) at 100%, followed by 16.67% in male mice with E2 administration (*n* = 6), and none observed in either sham-operated female mice (*n* = 3) or in female mice post-ovariectomy (*n* = 6). (**B**) VT/VF could be induced in all sham-operated male mice (*n* = 4), followed by 83.33% in male mice given E2 (*n* = 6), 33.33% in sham-operated female mice (*n* = 3), and 50% in female mice post-ovariectomy (*n* = 6). M, sham-operated ApoE^−/−^ male mice; M + E, ApoE^−/−^ male mice with 17β-estradiol (E2) injection; F, sham-operated ApoE^−/−^ female mice; OVx, ApoE^−/−^ female mice with ovariectomy.

In order to elucidate the mechanisms that link the male gender, elevated plasma L5 levels, and increased risk of early VT/VF in STEMI patients, we conducted ex vivo ischemic-reperfusion experiments on eight-week-old ApoE^−/−^ mice. These mice were given a high-fat diet for eight weeks to stimulate atherogenesis. The outcomes indicate that spontaneous VT/VF was observed in 100% of the sham-operated male mice (*n* = 4), which was reduced to 16.67% in male mice administered with 17β-estradiol (E2) (*n* = 6). No spontaneous VT/VF was detected in sham-operated female mice (*n* = 3) or in ovariectomized female mice (*n* = 6) (Figure 2). VT/VF was inducible in all sham-operated male mice (100%), 83.33% of male mice injected with 17β-estradiol (E2), 33.33% of sham-operated female mice, and 50% of ovariectomized female mice (Figure 2).

**Figure 3:**
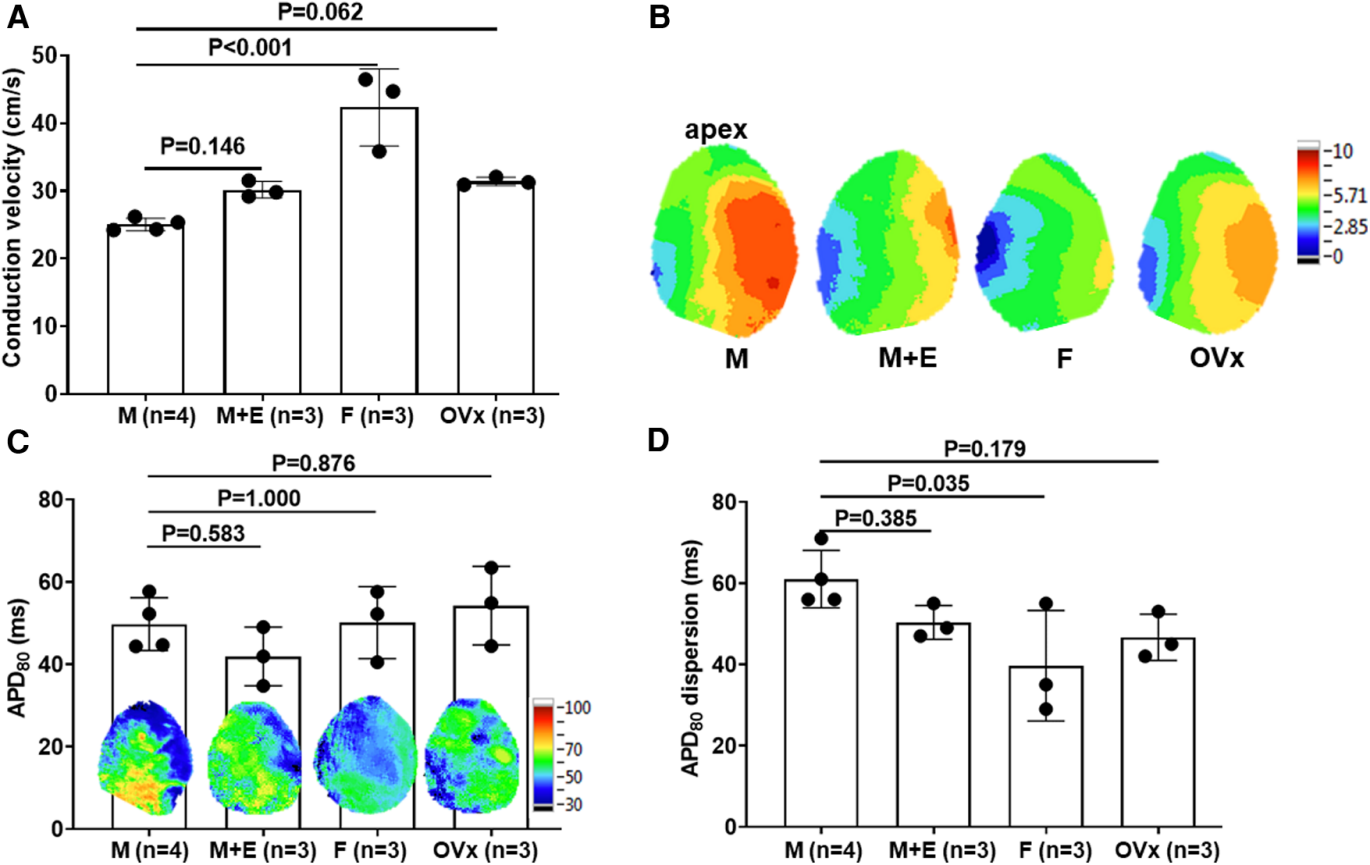
Optical mapping analysis in an ex vivo ischemia-reperfusion mouse model. (**A**) Optical mapping revealed a significant delay in heart conduction velocity in male mice (*n* = 4) relative to female mice (*n* = 3) in whole heart specimens. This slowed conduction was partially rectified in male ApoE^−/−^ mice that had undergone estrogen pretreatment (*n* = 3). (**B**) Showcases representative conduction map instances for the four study groups. (**C**) Comprehensive action potential duration (APD) mapping revealed similar APD values between male and female mice in whole heart preparations. We calculated the average of all the pixels to report APD80 values. (**D**) Sham-operated male mice (*n* = 4) displayed a significant increase in APD dispersion compared to female controls (*n* = 3), a deviation that was partially counteracted in male ApoE^−/−^ mice that received estrogen pretreatment (*n* = 3). For the comparison of more than two groups, we utilized one-way analysis of variance (ANOVA) along with Tukey's *post hoc* analysis to account for multiple comparisons. The group abbreviations are the same as those in Figure 2.

In an optical mapping analysis, there was a significant decrease in the conduction speed of the sham-operated male mice hearts as compared to the female mice (25.01 ± 0.93 vs. 42.32 ± 5.70 cm/s, *P* < 0.001). However, this deceleration in conduction speed was partially mitigated in the male ApoE^−/−^ mice that received estrogen pre-treatment (25.01 ± 0.93 vs. 30.16 ± 1.21 cm/s, *P* = 0.146) (Figure 3). During the evaluation of the global action potential duration (APD) mapping, the APD_80_ was found to be similar across all four animal groups (49.74 ± 6.42, 41.89 ± 7.13, 50.10 ± 8.73, and 54.23 ± 9.53 ms, *P* = 0.338). However, significant APD_80_ dispersion was observed in sham-operated male mice in comparison to their female counterparts (male: 61.00 ± 7.07, male with E2 pre-treatment: 50.33 ± 4.16, female control: 39.67 ± 13.61, female post-ovariectomy: 46.67 ± 5.69 ms, *P* < 0.001) (Figure 3).

**Figure 4:**
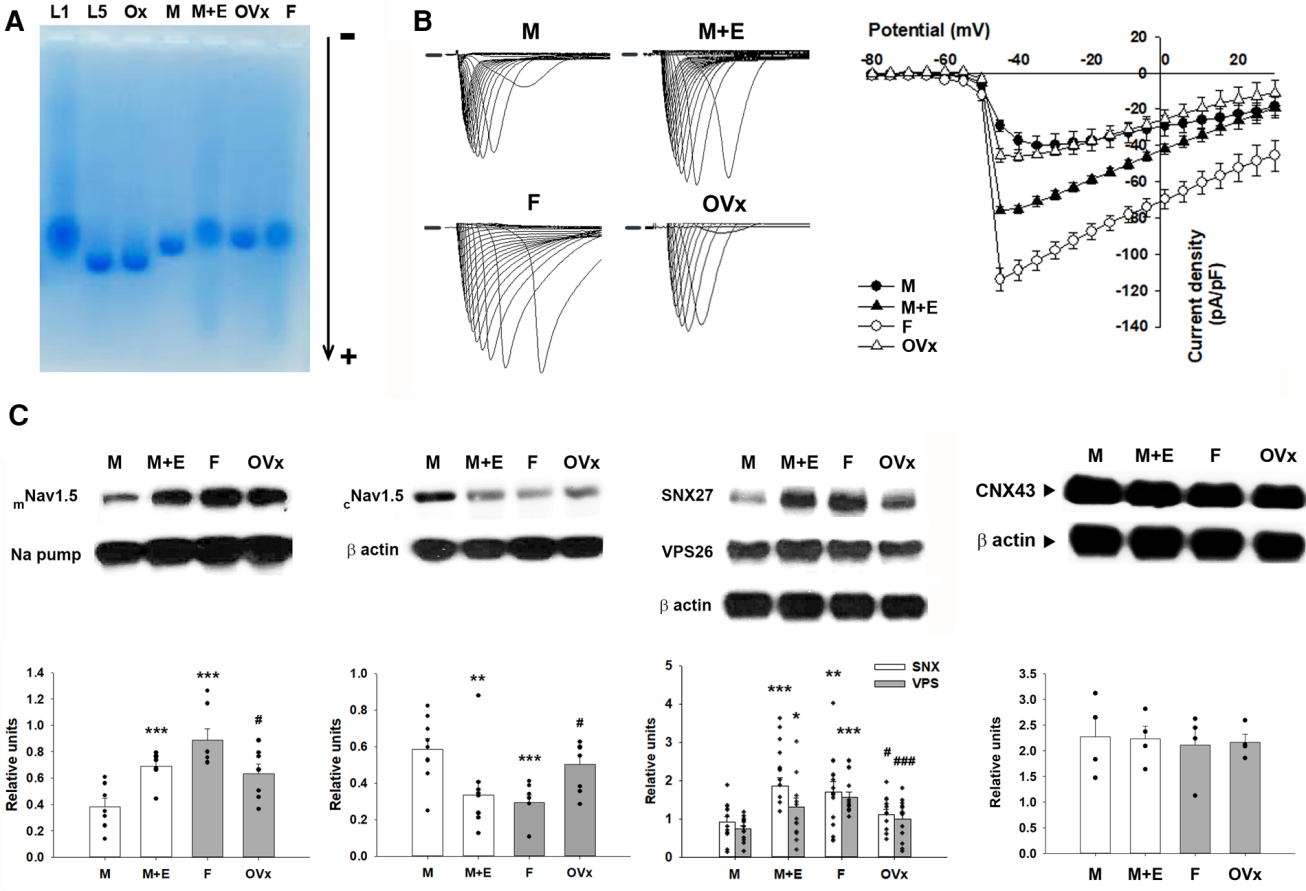
Comparative analysis of LDL electronegativity, sodium currents, and protein levels across four animal groups. (**A**) Representative outcomes of agarose gel electrophoresis display the relative electronegativity of LDL extracted from male and ovariectomized female mice, which contrasts with those from female and E2-administered male mice. L1 served as a negative control, whereas L5 and oxidized LDL acted as positive controls. (**B**) Ventricular myocytes isolated from male mice demonstrated a noticeably reduced sodium current density compared to their female counterparts. This reduction was partially alleviated in male ApoE^−/−^ mice subjected to estrogen pretreatment. (**C**) The diminished sodium current density was linked to a reduced membranous Nav1.5 protein expression and increased cytosolic Nav1.5 levels in male mice. In investigating the regulation of membranous/cytosolic fraction of Nav1.5 by the retromer, we found a significant decrease in total expression of Sorting Nexin 27 (SNX27) and Vacuolar Protein Sorting 26 (VPS26) in male mice compared to their female littermates. Connexin 43 (CNX43) protein expression remained consistent across all groups. Quantification of protein expression is expressed relative to the expression of the sodium pump (membranous internal loading control) or β-actin (cytosolic internal loading control). Quantitative analysis was conducted with a minimum sample size of *n* = 4 for each group, with individual samples represented by each black dot. For the comparison of more than two groups, we utilized one-way analysis of variance (ANOVA) along with Tukey's *post hoc* analysis to account for multiple comparisons. *P < 0.05, **P < 0.02, ***P < 0.01 in comparing with the “M” group. ^#^*P* < 0.05, ^###^*P* < 0.01 in comparing with the “F” group. The group abbreviations align with those in Figure 2.

The electronegativity of plasma LDL was assessed through gel electrophoresis analyses, with L5 and oxidized LDL serving as positive controls and L1 as a negative control. In a native agarose gel, LDL from male and ovariectomized female mice migrated towards the anode more rapidly than the LDL from the female or E2-administered male mice (Figure 4). This suggests that LDL from male and ovariectomized female mice was more electronegative than that from female or E2-administered male mice. These results imply a heightened vulnerability to VT/VF related to ischemic-reperfusion in the male gender, associated with LDL electronegativity.

To understand the pronounced slowing of conduction observed in the hearts of sham-operated ApoE^−/−^ male mice, we compared the sodium current densities across all four groups of mice (Figure 4). The results illustrated a significantly reduced sodium current density in the isolated ventricular myocytes of sham-operated male mice (−29.20 ± 3.04 pA/pF, *n* = 6; *P* < 0.001). This was followed by the ovariectomized female mice (−45.32 ± 3.83 pA/pF, *n* = 6; *P* < 0.01), and male mice administered with E2 (−75.78 ± 1.98 pA/pF, *n* = 6; *P* < 0.001), relative to the sham-operated female mice (−114.05 ± 6.41 pA/pF, *n* = 6) at 45 mV. Among these groups, the sodium current in the myocytes of sham-operated female mice was significantly larger than that in the myocytes of ovariectomized female mice (*P* < 0.001).

Quantitative protein expression was performed through western blotting experiments (Figure 4). Analysis of cardiac tissues showed a significant downregulation of the membrane fraction, accompanied by an upregulation of the cytosolic fraction of the sodium channel (Nav1.5) in male mice, compared to their female counterparts (0.38 ± 0.06 vs. 0.89 ± 0.09; *P* < 0.01 and 0.59 ± 0.06 vs. 0.29 ± 0.04; *P* < 0.01, respectively). The administration of E2 in male mice was able to restore the membrane fraction of Nav1.5 (0.69 ± 0.05; *P* < 0.01) and subsequently decrease its cytosolic fraction (0.34 ± 0.07; *P* = 0.02) relative to the male control group. Conversely, female mice subjected to ovariectomy exhibited a decreased membrane fraction of Nav1.5 (0.63 ± 0.07; *P* = 0.04) and an increased cytosolic fraction of Nav1.5 (0.50 ± 0.05; *P* = 0.04) compared to the female control group. Connexin 43 (CNX43) protein expression which also mediates cellular conduction were similar in the four groups.

In an attempt to examine the role of retromer-mediated regulation of membranous/cytosolic fractions of Nav1.5, we observed that the total expression levels of Sorting Nexin 27 (SNX27) and Vacuolar Protein Sorting 26 (VPS26) were significantly lower in male mice compared to their female counterparts (0.91 ± 0.15 vs. 1.70 ± 0.28; *P* = 0.02 and 0.74 ± 0.09 vs. 1.57 ± 0.13; *P* < 0.01, respectively). Similar to the previous observation, the introduction of E2 in male mice resulted in the restoration of both protein abundances (1.86 ± 0.22; *P* < 0.01 and 1.31 ± 0.23; *P* = 0.03). Conversely, the ovariectomy in female mice led to a reduction in the abundances of these proteins (1.12 ± 0.14; *P* = 0.04 and 1.00 ± 0.16; *P* < 0.01) when compared to their respective gender controls.

## Discussion

To our understanding, this study provides the initial evidence of a gender-specific risk for early VT/VF, combining a hospital-based patient dataset with a relevant animal model to investigate the underlying mechanisms. The critical discoveries of this research include: (1) an observed male predominance coupled with elevated L5 levels in STEMI patients who experienced early VT/VF, based on a comprehensive dataset from hospital-based STEMI patients; (2) a notably higher incidence of spontaneous VT/VF in a male ApoE^−/−^ animal model, compared to their female counterparts; (3) slower conduction velocity and increased APD80 dispersion in male animal models compared to females; (4) a reduction in the ratio of membranous to cytosolic sodium channel protein, along with a decrease in sodium currents in males compared to females, which is partially attributed to the retromer-mediated recycling pathway; (5) Collectively, these findings provide a plausible explanation for the observed gender-based disparities in early VT/VF susceptibility among STEMI patients.

### Gender-specific risk of early VT/VF in STEMI patients

The occurrence rate of early VT/VF within the first 48 h following the onset of STEMI has been reported to range from 5.6% to 11.6%, based on registry and hospital-sourced data (1, 2, 19). Within studies encompassing all myocardial infarction cases, including STEMI and non-STEMI, male sex has consistently demonstrated a higher risk of early VT/VF across various ethnic populations (2, 3, 20). In a nationwide case-control study conducted by Jabbari et al. (2), the incidence rate of early VF within the first 12 h of STEMI, prior to PPCI, was found to be 11.6% with a male predominance of 86%. A study by Hai et al. (20) indicated that 7.6% of patients with STEMI, non-STEMI, or unstable angina developed early VT/VF within 48 h of acute coronary syndrome onset. The male proportion was higher among early VT/VF patients in a Chinese population. Echoing these findings, our study identified 255 (8.6%) patients who developed early VT/VF within 48 h of STEMI onset from a pool of 2,964 consecutive STEMI patients. In our cohort of STEMI patients with early VT/VF, the male proportion was higher (87%) than in those without early VT/VF. Furthermore, we noticed a trend towards younger age in STEMI patients with early VT/VF (58 vs. 61 years old), corroborating with earlier reports (1, 2, 19).

### Male predominance in out-of-cardiac arrest (OHCA) populations

The observation of male predominance in VT/VF or initial shockable rhythm associated with Out-of-Hospital Cardiac Arrest (OHCA) has been consistently affirmed in global studies (15, 21–25). Agusala et al. (25), in their analysis of data from the Resuscitation Outcomes Consortium's Continuous Chest Compressions trial cohort, found that 83% of OHCA patients who presented with an initial shockable rhythm also exhibited electrocardiographic ST elevation. This contrasts with 63% of OHCA patients with ST elevation but without a shockable rhythm. Furthermore, Havranek et al. (23) reported a higher proportion of male patients presenting a shockable rhythm (90% vs. 71%) in witnessed OHCA of presumed cardiac origin, when compared with those presenting non-shockable rhythms. These findings are corroborated by our previous research, wherein we determined male sex, age below 65 years, public location of arrest, and witnessed status as independent predictors of VT/VF rhythm in a city-wide non-selective registry population of 1,629 presumed cardiogenic OHCAs. The male predominance in VT/VF OHCA was further reinforced in a multicenter cohort study (22). Collectively, these findings endorse the hypothesis that male patients exhibit greater susceptibility to VT/VF related sudden cardiac death, regardless of whether the context is STEMI or OHCA, and this is consistent across different ethnic groups.

### Low-density lipoprotein electronegativity and ventricular tachyarrhythmias

Elevated LDL levels at one week and three months post-STEMI have been identified as directly proportional with the incidence of early VT/VF, and they can independently predict its development (10). Statin use, which lowers LDL levels, has been demonstrated to confer protective effects against VT/VF occurrence after acute myocardial infarction (26). Moreover, patients with acute coronary syndrome who had been on statins were significantly less prone to in-hospital VT/VF (27). However, Riahi et al. (28) found limited evidence supporting the antiarrhythmic effects of statins, or any correlation between plasma lipids and lipoproteins and severe ventricular arrhythmias in coronary artery disease patients with an Implantable Cardioverter Defibrillator (ICD). Intriguingly, our current study revealed similar lipid profile levels, including total cholesterol, triglycerides, and LDL, between the two patient groups, but a lower high-density lipoprotein level in the VT/VF group. In a subpopulation of our study, we found that STEMI patients with early VT/VF complications exhibited significantly higher plasma levels of L5, the most electronegative subclass of LDL, compared to those without early VT/VF. These contrasting results may be attributed to differences in patient populations under study, but it's also plausible that the most electronegative LDL fraction, L5, may account for the link between dyslipidemia and susceptibility to VT/VF. Exploring L5's role in ventricular tachyarrhythmias, we previously demonstrated that L5 might directly modulate the electrophysiological properties by altering sarcolemmal ionic currents through LOX-1 signaling in ventricular cardiomyocytes, according to *in vivo* and *in vitro* experiments, which could provide insight into a potential role of L5 in early VT/VF occurrence in STEMI patients (29).

### Sexual dimorphism associated reduction of sodium currents

To unravel the mechanisms underpinning the higher susceptibility of males to early VT/VF following STEMI, we employed a pertinent ApoE^−/−^ mouse model subjected to ex vivo ischemia-reperfusion, mimicking the reperfusion injury experienced by STEMI patients undergoing primary percutaneous coronary intervention therapy (30). Our findings revealed a greater predisposition towards VT and VF in male ApoE^−/−^ mice post-ischemia-reperfusion injury, aligning with the observed male dominance in clinical scenarios involving STEMI patients receiving reperfusion therapy. Our study suggests that electrophysiological alterations are instrumental in this gender disparity, as evidenced by slower cardiac conduction velocity, augmented dispersion of action potential duration (APD), and diminished sodium current density due to decreased expression of the Nav1.5 protein in the membranes of male mice.

To delve deeper into the mechanisms contributing to these electrophysiological alterations, we identified that the injection of E2 partially reversed conduction slowing in male mice. This coincided with modifications to sodium currents and the membranous expression of Nav1.5 proteins. In harmony with this, female mice that underwent ovariectomy demonstrated adverse electrophysiological remodeling, marked by a reduction in sodium currents, conduction deceleration, and decreased sarcolemma Nav1.5 protein levels. These findings accord with previous research highlighting the modulation of ion channel function and myocardial electrophysiology by estrogens and androgens (31, 32). Furthermore, we underscore the importance of identifying the signaling pathways and molecular intermediaries responsible for the disparate regulation of Nav1.5 expression and functionality in male and female mice. In our study, we observed a positive correlation between elevated levels of LDL electronegativity and an increased propensity for VT/VF, both in human subjects and our animal model. Previous work revealed a gender-specific impact of the most electronegative LDL, L5, on vascular athero-inflammatory modifications (33). Also, we have shown that L5 can directly influence electrophysiological properties by altering sarcolemma ionic currents through LOX-1 signaling in ventricular cardiomyocytes, according to both *in vivo* and *in vitro* experiments (29). These observations resonate with past findings associating dyslipidemia with elongated action potential duration (APD), extended QTc intervals, enhanced repolarization dispersion, and increased ventricular fibrillation susceptibility in a rabbit model (34). Further investigations are indeed required to discern the role of sex hormones in regulating Nav1.5 protein expression and functionality. Such research could yield a deeper comprehension of the noted gender disparity in VT/VF susceptibility, potentially guiding the development of specialized therapies to counteract the heightened risk of VT/VF in males.

Nav1.5's final three residues, Ser-Ile-Val (SIV), form a PDZ (postsynaptic density protein-95, Discs-large, Zona-occludens-1) binding motif, crucial in controlling the degradation of Nav1.5 at the membrane level (35). SNX27, featuring a PDZ domain, operates as a structural platform for organizing multiple proteins. Notably, the SNX27-incorporated retromer is a key regulator of the recycling process from endosome to plasma membrane for transmembrane cargos carrying a PDZ-binding motif. Herein, cardiomyocytes' membranous receptor proteins can affiliate with SNX27, thereby avoiding entry into the lysosomal pathway and instead recycling through the endosome (36). The PDZ domain of SNX27 directly couples with VPS26 to form the SNX27-retromer, which is both necessary and sufficient to hinder the entry of SNX27-dependent cargo into the lysosomal pathway (37). In the present study, we observed lower membranous expression of Nav1.5 alongside a concomitant reduction of the SNX27-VPS26-retromer. This occurrence might partially explain the functional decline of sodium current density. Nevertheless, additional research is essential to clarify the precise mechanisms underpinning the observed gender disparity in the regulation of sodium currents within this ischemia-reperfusion model.

## Limitations

Our research has several limitations worth noting. Firstly, despite establishing the clinical correlation between younger male patients and an elevated risk of VT/VF SCD, it is essential to conduct further research enlisting patients with genetic data. Incorporation of clinical and ECG risk factors, such as the SACAF score (22), would aid in creating a comprehensive risk score to pinpoint STEMI patients with a high predisposition for primary VT/VF sudden death. Secondly, our findings, grounded in an ex vivo mouse model, warrant validation in larger animal models. Such a move would substantiate the translational potential of our observations and their relevance in clinical contexts. Third, the human sample size for measuring L5 was limited because the measurement of L5 in the human sample was constrained by technical challenges and limitations. Specifically, for analyzing the percentage of L5, a minimum of 30 ml whole blood samples within a 72-h timeframe is necessary for a subset of STEMI patients. Enrolling patients into our study, particularly those with VT/VF (with an incidence rate of less than 10% in Taiwan), presents a significant challenge. Lastly, although we delineated the association between LDL electronegativity and the susceptibility of VT/VF in males and in our animal model, a deeper understanding of the molecular and electrophysiological mechanisms governing the sex-specific differences in VT/VF predisposition is required. This comprehension could provide the foundation for devising novel therapeutic strategies to mitigate the risk of VT/VF in STEMI patients, particularly those male patients at higher risk.

## Conclusions

Our research underscores that male patients with STEMI who exhibit early VT/VF possess elevated levels of L5, the most electronegative LDL subclass. This gender-based discrepancy in susceptibility to early VT/VF could potentially be attributed to impaired sodium channel trafficking, possibly associated with enhanced LDL electronegativity. This insight could guide future research efforts to devise targeted therapeutic approaches for improving patient outcomes, especially those at a heightened risk of fatal ventricular arrhythmias post-STEMI.

## Data Availability

The raw data supporting the conclusions of this article will be made available by the authors, without undue reservation.
